# A rare and severe complication after minimally invasive esophagectomy: First case of a left-sided tension pneumothorax caused by intrathoracic perforation of the herniated transverse colon. Case report and literature review

**DOI:** 10.3389/fgstr.2023.1109999

**Published:** 2023-03-06

**Authors:** Karim Mostafa, Carmen Wolf, Johannes Austrup, Frederike Franke, Olav Jansen, Marcus Both, Patrick Langguth

**Affiliations:** ^1^ Department of Radiology and Neuroradiology, University Hospital Schleswig-Holstein, Kiel, Germany; ^2^ Department of General, Visceral, Thoracic and Childrens’ Surgery, University Hospital Schleswig-Holstein, Kiel, Germany

**Keywords:** minimally invasive esophagectomy, intrathoracic colonic perforation, intrathoracic closed loop obstruction, postesophagectomy diaphragmatic herniation, tension pneumothorax

## Abstract

Nowadays, a minimally invasive surgical approach is increasingly being chosen to treat distal esophageal tumors. Here, postoperative hiatal herniation has been identified as a potentially severe complication. In such cases, it is still not known whether surgical or conservative treatment is preferable. In this report, we elaborate the case of a 62-year-old male patient who presented at our emergency department with severe chest pain. This patient had undergone minimally invasive esophagectomy with gastric pull-up 2 years prior to this event. Emergency computed tomography revealed a left-sided tension pneumothorax based on transhiatal herniation of the transverse colon causing an intrathoracic closed-loop obstruction with subsequent perforation. Immediate surgical treatment was initiated and the transverse colon could be successfully repositioned and resected. Nevertheless, the patient died due to postoperative septic shock in the setting of fecal peritonitis, mediastinitis, and pleuritis within 48 hours after surgery. We provide a detailed description of this rare case and provide a review of the literature concerning intrathoracic colonic herniations.

## 1 Introduction

Nowadays, curative treatment of esophageal tumors can be achieved by neoadjuvant chemotherapy followed by minimally invasive surgery with resection of the esophagus and subsequent gastric pull-up and cervical anastomosis. Known common complications after this technically challenging procedure include pneumonia, pleural empyema or fistula, and anastomotic insufficiency. In recent years, postoperative hiatal hernia or “postesophagectomy hiatal prolapse” has been identified as another potentially severe complication. In acute settings, patients presenting with such a hernia show mortality rates as high as 20% (1, 2). Such hernias frequently develop within the first year after surgery and are predominantly located in the left hemithorax. The transverse colon is reported to be the most commonly herniated abdominal organ after esophagectomy (2). Indeed, such a hernia likely develops as a result of perioperative manipulation of the esophageal hiatus and is additionally provoked and facilitated by the positive pressure in the abdominal cavity and negative pressure in the thoracic cavity.

We describe the case of an adult patient with a transhiatal colonic hernia after minimally invasive esophagectomy. The colonic hernia was complicated by a closed-loop obstruction at the level of the hiatus. Subsequently, the herniated colon perforated intrathoracically and a left-sided tension pneumothorax developed in this patient. To our knowledge, only one other case characterized by such a herniation with closed-loop obstruction and intrathoracic perforation has been reported so far; however, that one differs from ours in site of the perforation, which was located in the mediastinum without pleural involvement or pneumothorax (3). Furthermore, we provide a review of the literature dealing with such intrathoracic colonic herniations, both spontaneously and in a post esophagectomy setting.

## 2 Materials, methods and results

### 2.1 Patient information and clinical findings

A 62-year-old man presented at our emergency department with severe thoracic pain radiating to his left shoulder. Previously, the patient had collapsed at home and contacted emergency services himself. On admission, the patient was tachycardic at a heart rate of 110 beats per minute and a blood pressure of 140/80 mmHg. Body temperature was 36.9°C. The patient complained of shortness of breath, with an oxygen saturation of 85% at room air. During examination, the patient still complained about severe thoracic pain, NRS 9/10, that did not respond to the medication he received. Recapillarization time was elongated to 5 seconds. Breath sounds were absent over the left hemithorax. Last bowel movement was reported to have happened four days prior to this event. The patient’s past medical history included an esophagectomy with gastric pull-up due to distal esophageal cancer that had been performed 2 years ago. Perioperatively, a port catheter had been implanted in his left subclavian vein for administration of chemotherapy.

### 2.2 Diagnostic assessment

**Figure 1:**
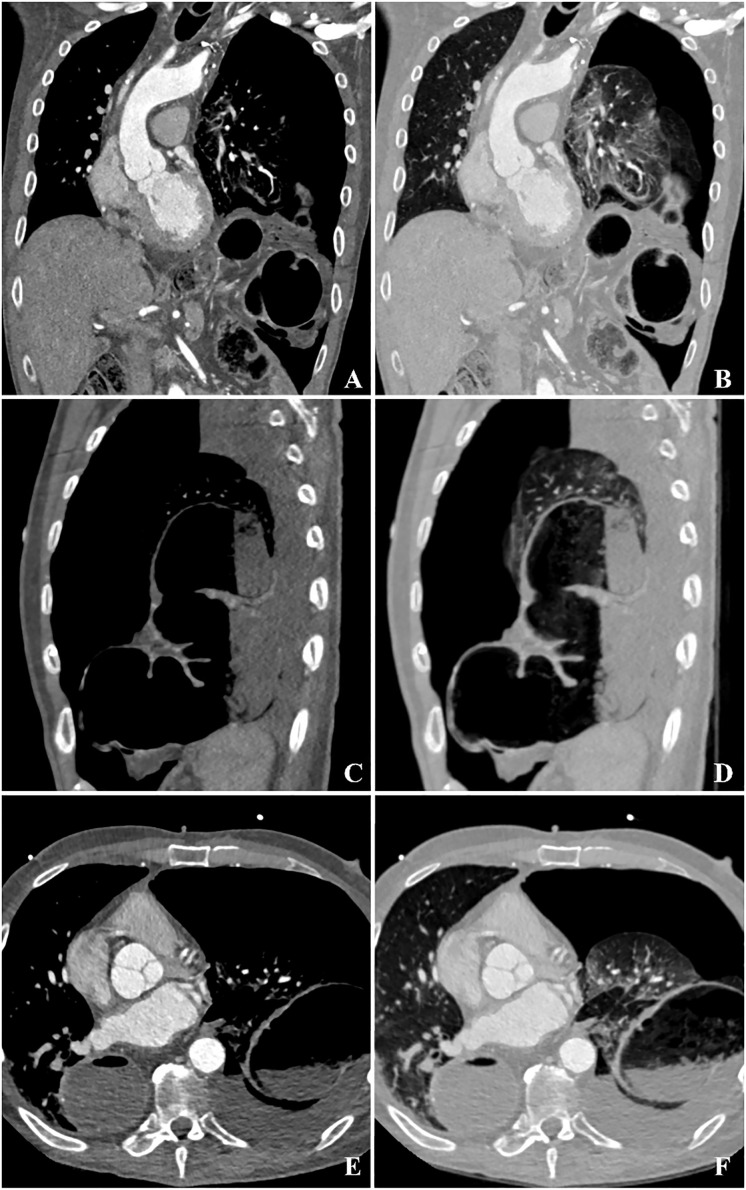
Triple-rule-out CT imaging of the described patient in coronal **(A, B)**, sagittal **(C, D)**, and transverse **(E, F)** reconstructions. **(A, B)** show a left-sided tension pneumothorax with mediastinal displacement to the left. In the hiatal area, torsion of the transverse colon and mesocolon reveals a closed-loop obstruction. A density-increased pleural effusion and dilated transverse colon in the left hemithorax can be seen **(C-F)**, suggesting a colonic perforation as cause for pneumothorax.

**Figure 2:**
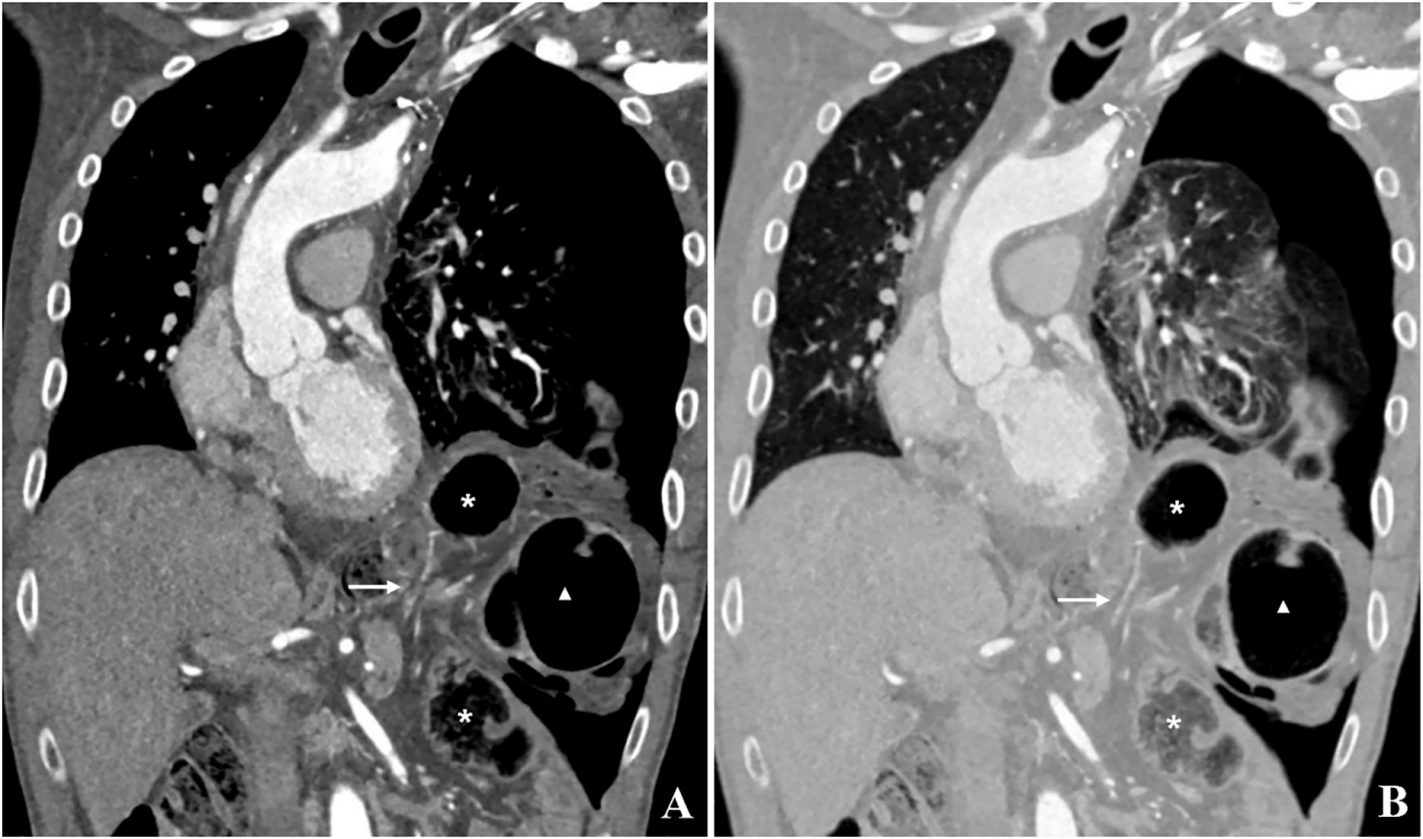
**(A, B)** CT image in coronal reconstruction. A diaphragmatic defect with twisted colon and mesocolon entering and exiting the left hemithorax can be seen. Due to twisting of the colon at the level of the diaphragmatic defect (Arrow) with subsequent closed-loop obstruction, the herniated intrathoracic colon loops are dilated (Star and Triangle). Arrow, Diaphragmatic defect; Star, Aboral part of the transverse colon; Triangle, Oral part of the transverse colon.

At admission, core laboratory values included slightly elevated C-reactive protein (5,61 mg/l, reference 0.0 – 5.0 mg/l). Point-of-care abdominal ultrasound was unresolving due to bowel gas overlay. Owing to the patient’s progressively worsening pain that did not respond to medication, computed tomography (CT) was performed, since at this point a major aortic pathology was being suspected. Imaging showed a left-sided tension pneumothorax with density-increased pleural effusion and a dilated transverse colon in the left hemithorax, which had herniated through the esophageal hiatus (**Figure 1**). At the hiatal level, torsion of the transverse colon and mesocolon was seen entering and exiting the thoracic cavity, confirming the diagnosis of a closed-loop obstruction (**Figure 2**). This caused an intrathoracic colon perforation, subsequently producing a tension pneumo- and fecothorax. In addition, both adrenal glands were hyperattenuated in the arterial and venous phase, suggesting septic shock (4). Furthermore, CT imaging revealed multiple thrombi along the still-inserted port catheter, with the tip in the patient’s left brachiocephalic vein. Occlusions of the right subclavian and internal jugular vein were already known from previous imaging studies.

### 2.3 Therapeutic intervention

Based on the clinical findings and the patient’s condition, possible therapeutic approaches were discussed in an interdisciplinary consultation. As the next steps, a chest tube was to be placed in the emergency department, followed by surgical reposition and resection of the perforated transverse colon. After informing the patient of his condition and careful explanation of the therapeutic options, he agreed to the proposed procedures. Antibiotic treatment with intravenous meropenem was initiated in the emergency room and a 24-French chest tube was placed into the left hemithorax, draining air and stool from the pleural space. In the operating room, a laparotomy was then performed and the herniated transverse colon was repositioned into the abdomen. The perforated segment of the colon was resected. The esophageal hiatus was narrowed, taking care not to compromise the lumen of the gastric pull-up. The procedure was completed by extensive left-sided pleural lavage to reduce the risk of fecal pleuritis.

### 2.4 Results and outcome

Perioperatively, sepsis began to develop and the patient’s circulatory system became unstable, suggesting septic shock. In addition, the patient developed signs of upper inflow-congestion, which was confirmed postoperatively on repeat CT examination and which was most likely due to the aforementioned thrombi along the port catheter and its tip obstructing the superior vena cava and resulting in venous congestion. At this point, it was assumed that the perioperative fluid resuscitation had destabilized the already restricted drainage capacity of the craniocervical venous system. Venous stenting was discussed but, owing to the recent fecal pleural effusion, no action was taken. Forty-eight hours after surgery, the patient died of cardiac failure in the framework of septic shock due to fecal peritonitis, mediastinitis, and pleuritis.

## 3 Discussion

In this report, we present a patient with an intrathoracic colonic closed-loop obstruction and subsequent perforation and tension pneumothorax caused by herniation of the transverse colon through the esophageal hiatus after minimally invasive esophagectomy and gastric pull-up. To the best of our knowledge, this is the first case report ever to describe this etiology for a tension pneumothorax. In the upcoming sections, we provide a review of the literature on transhiatal herniations in patients after esophagectomy and their associated complications and we discuss potential treatment approaches.

### 3.1 Postesophagectomy diaphragmatic hernias

Nowadays, with the advent of minimally invasive surgical procedures, complications such as hiatal hernias have become more common. A recent review reports an incidence of up to 6% for diaphragmatic transhiatal herniations after minimally invasive esophagectomy (5). A study by Fuchs et al. investigating 39 patients with transhiatal herniation after minimally invasive esophagectomy between 2003 and 2017 showed that, most commonly, the transverse colon herniates into the left hemithorax, as in our patient (2).

The genuine esophageal hiatus is most commonly associated with various types of gastric hernias. In our case, however, we find the rare entity of an isolated colonic hernia that prolapsed through the esophageal hiatus, leaving the small bowel in the abdominal cavity. In the past, such isolated colonic hernias were not considered a common finding in patients who had undergone esophagectomy and gastric pull-up. Tabira et al. reported in 2004 that, up to that point, only four cases of isolated colonic hernias had been described in the literature (6–10). Another five similar cases of isolated colonic hernias were reported between 2008 and 2016; however, none of these cases were associated with an esophagectomy (**Table 1**). Indeed, only one similar case report of intrathoracic colonic herniation with mediastinal perforation following esophagectomy has been published in the literature so far (3). The patient presented in that specific case developed symptoms 3 years after laparoscopic esophagectomy, but was not complaining of any pain at admission. Diagnostic imaging revealed an incarcerated herniation of the transverse colon in the right side of the mediastinum, and surgical treatment was immediately initiated. Intraoperatively, perforation was evident, and the affected colon was resected. The perforation was limited to the posterior mediastinum, which differs significantly from our case in which the perforation involved both the mediastinum and the pleura.

Table 1Overview of the literature on transhiatal colonic herniation, with and without prior esophagectomy.

**Table:** 

Literature overview – Reports of diaphragmatic herniation after esophagectomy								
Author, Year	Title	Article Type	Esophagectomies (MIC)	Diaphragmatic hernia	Hernia involving colon		Isolated colonic hernia	Perforation
Matthews et al. (1)	Diaphragmatic herniation following esophagogastric resectional surgery: An increasing problem with minimally invasive techniques?	Cohort study	506 (73)	31	30		N.r.	N.r.
Fuchs et al. (2)	Transdiaphragmatic herniation after transthoracic esophagectomy: an underestimated problem	Cohort study	39 (39)	39	34		N.r.	N.r.
Lee et al. (5)	Increased risk of diaphragmatic herniation following esophagectomy with a minimally invasive abdominal approach.	Systematic review	17052 (432)	533	N.r.		N.r.	4
Brenkmann et al. (11)	Hiatal Hernia After Esophagectomy for Cancer	Cohort study	657	45	37		N.r.	N.r.
Konno-Kumagai et al. (3)	Transverse colon perforation in the mediastinum after esophagectomy: A case report.	Case report	1	1	1		N.r.	1

**Table:** 

Reports of isolated transhiatal colonic herniation without prior esophagectomy								
Author, Year	Title	Article type	Complicating factors			Intrathoracic perforation with pneumothorax		
Curtis et al. (6)	Isolated Herniation of the Colon Through the Esophageal Hiatus: Case Report	Case report	None			No		
Felsher et al. (7)	Isolated trans-hiatal colonic herniation	Case report	None			No		
Itabashi et al. (8)	A Case of Esophageal Hiatal Hernia with Incarcerated Transverse Colon	Case report	Intraabdominal perforation			No		
Sonderstrup et al. (9)	Life-threatening obstruction of the airway caused by a herniated colon loop	Case report	None			No		
Tabira et al. (10)	Isolated colonic hernia through the esophageal hiatus.	Case report	None			No		
Ooi et al. (12)	Laparoscopic repair of gastric volvulus secondary to transverse colon diaphragmatic hernia	Case report	Gastric volvulus			No		
Yildiz et al. (13)	Isolated Intrathoracic Hiatal Herniation of the Twisted Sigmoid Colon	Case report	Colonic volvulus			No		
Altinkaya et. al (14)	MDCT Diagnosis of Isolated Colonic Hernia Through the Esophageal Hiatus	Case report	None			No		
Self et al. (15)	Isolated colonic hernia through the oesophageal hiatus causing gastric outlet obstruction	Case report	Gastric outlet obstruction			No		

In the upper part of the table, reports of transhiatal colonic herniations after esophagectomy are shown. Note that although the number hernias is high, perforations are reported to have occurred in only 5 patients; however, none of the patients with perforation was reported to have pneumothorax.

In the lower part of the table, an overview of the literature on transhiatal colonic herniations without prior esophagectomy is given. Note that there are only 9 case reports available describing this rare entity. This further supports the claim that colonic herniation is a condition most likely affecting patients after esophagectomy. MIC, Minimally invasive Esophagectomy; N.r, Not reported.

### 3.2 Management strategies and assessment

**Figure 3:**
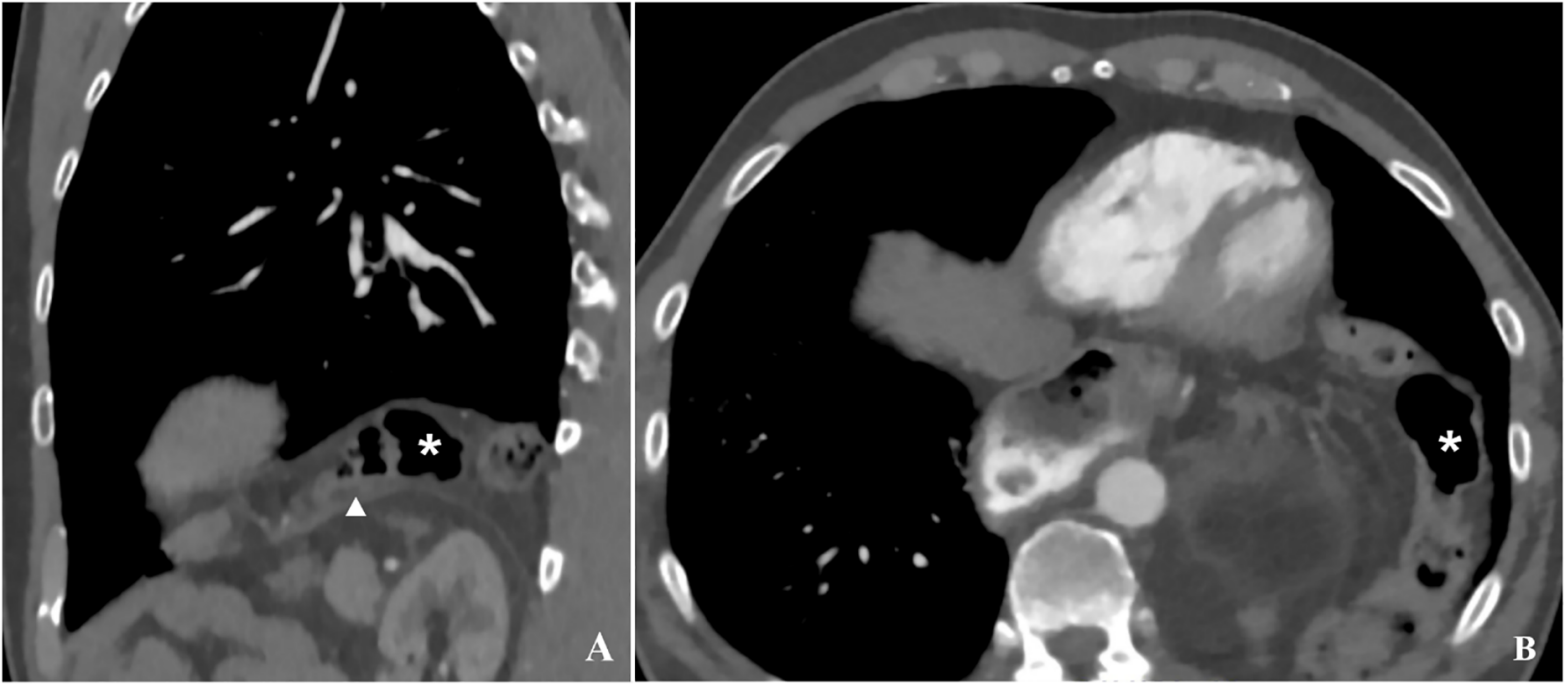
Latest available follow-up CT imaging prior to the patient presenting with acute symptoms in sagittal **(A)** and transverse **(B)** reconstruction. At the base of the thorax, the transverse colon (star) can be seen directly above the diaphragm, suggesting a larger scale hernia. This is difficult to see in axial reconstructions **(B)**, however it becomes evident in sagittal reconstructions **(A)**. The triangle on image B marks the left hemidiaphragm.

Our patient had surgery in June of 2020, and a small intrathoracic hiatal hernia of the transverse colon and omentum at the base of the left hemithorax was retrospectively already seen in the CT imaging immediately after surgery (**Figure 3**), and the hernia showed continuous progression at the follow-up CT examinations. Small asymptomatic hernias can be controlled with a wait-and-see approach as well as treated surgically at an early stage (3, 11). However, symptomatic hernias are known to be associated with a high mortality of up to 20% (1, 2). In our patient, early surgical repair would probably have been beneficial despite the absence of symptoms.

Patients who develop acute symptoms after esophagectomy must be examined promptly. Here, in addition to pain characteristics and pain localization, symptoms such as dyspnea and chest pain in particular should be considered red flags for any complication associated with the surgery, including transhiatal herniation (16). Diagnostic imaging method of choice should be a two-phase CT with ECG gating to also rule out cardiovascular causes of severe chest pain. If available, current CT findings should be compared with prior imaging studies, as hernias may have already been present but not described. In our case, the presence of hernia on prior imaging studies was one of the decisive factors for making the correct diagnosis in a timely manner.

After diagnosis of an intrathoracic hernia, an interdisciplinary discussion on the best treatment should be conducted very soon, taking into account the patient’s general condition and complicating imaging findings: Perforation, pneumothorax, pleural effusion, size of the hernia, and presence of an obstruction should be considered. In such cases surgical intervention will be inevitable and outcomes will likely be fatal if a conservative approach is chosen. In the case of tension pneumothorax in the setting of post esophagectomy herniation, early chest tube placement should be performed prior to surgery. The exact surgical approach will vary from case to case, whereby clear radiological demonstration of the available imaging-is a crucial factor. For hernia replacement and resection of perforated bowel, an approach *via* median laparotomy will be feasible. Fecal pleural effusion requires pleural lavage, which in our case was performed *via* the previously placed chest tube. Taking the high degree of difficulty into account, such procedures should be conducted in specialized hospitals, where modern infrastructure as well as the specific surgical, radiological, and anesthesiologic expertise are available.

Perioperatively, broad-spectrum antibiotic treatment, fluid resuscitation, and circulatory support must be optimally managed to combat development of septic shock. This is crucial especially for the postoperative period, where further care needs to be provided in the setting of an intensive care unit where continuous monitoring is available. However, even if optimal care is ensured, outcomes will remain poor and mortality high. In our patient, in whom surgery was successful, the cause of death was most likely septic shock associated with complications of upper inflow obstruction.

## 4 Conclusion

Transhiatal intrathoracic bowel hernia is a known complication after minimally invasive esophagectomy, which can be asymptomatic for a long time. Here, we report the case of an intrathoracic herniation of the transverse colon with complicating closed loop obstruction, perforation, and development of a tension pneumothorax. Surgical management of such cases will remain the treatment of choice. However, even if optimal care is provided, outcomes are poor and often fatal, as it was in our patient. This report aims to raise awareness of postesophagectomy hiatal prolapse and to consider this unusual complication in the differential diagnosis of patients presenting with tension pneumothorax. It should provide an incentive for clinicians and radiologists to rule out a postesophagectomy diaphragmatic herniation in follow-up examinations or by imaging and, if caught early, to initiate surgical hernia repair in a timely manner.

## Data availability statement

The original contributions presented in the study are included in the article/supplementary material. Further inquiries can be directed to the corresponding author.

## Ethics statement

Ethical review and approval was not required for the study on human participants in accordance with the local legislation and institutional requirements. The patients/participants provided their written informed consent to participate in this study. Written informed consent was obtained from the individual(s) for the publication of any potentially identifiable images or data included in this article.

## Author contributions

CW and KM were involved in drafting the manuscript, image and table editing, reviewing the literature and managing the patients’ data. FF and JA were involved in the primary, operative and postoperative care of the patient as well as providing the patients clinical data. MB and OJ provided assessment of the first draft of the manuscript as well as managing of the manuscript editing process. PL was involved in manuscript and image editing as well as in reviewing of the first draft. All authors contributed to the article and approved the submitted version.
